# Value of a quality label and European healthcare professionals’ willingness to recommend health apps: An experimental vignette study

**DOI:** 10.1177/13591053241258205

**Published:** 2024-08-02

**Authors:** Ieva Biliunaite, Laurens van Gestel, Petra Hoogendoorn, Marieke Adriaanse

**Affiliations:** 1 Leiden University Medical Center, The Netherlands; 2 Leiden University, The Netherlands

**Keywords:** e-health, health apps, health behavior, healthcare professional, health care systems, recommending apps, socioeconomic status

## Abstract

This study aimed to evaluate healthcare professionals’ (HCPs’) willingness to recommend health apps presented with versus without the CEN-ISO/TS 82304-2 health app quality label. The study was an experimental vignette study describing 12 short hypothetical scenarios, with Label (absent vs present) as a between and Type of App (prevention vs self-monitoring vs healthcare) and Patient Socioeconomic Status (low vs high) as within-subjects factors. The main outcome measure was HCPs’ willingness to recommend apps. A total of 116 HCPs took part in the study. A significant main effect of the label was found. Further, HCPs were most willing to recommend self-management apps and more willing to recommend apps to high as opposed to low SES patients. However, the effect of the label did not differ between apps or according to patients’ SES. Results confirm that the quality label has potential for increasing willingness to changing HCPs’ recommendation behavior.

## Introduction

Healthcare professionals (HCPs; i.e. individuals who have completed specialized higher education studies to be able to provide preventive, curative, or other types of health services in the diagnosis and treatment of health problems (World Health Organization, 2019) have been previously identified as a very important, yet understudied group of stakeholders for adopting digital health technologies (Leigh and Ashall-Payne, 2019; Lupiáñez-Villanueva et al., 2020). In the healthcare setting, HCPs act as gatekeepers for patients in their access to relevant health apps (Lim et al., 2021). That is, patients may be directed to specific apps by their HCPs and may feel more motivated to use applications that are recommended by their HCPs (Jezrawi et al., 2022). However, currently, the extent to which HCPs actually recommend health apps is very limited. For example, in a recent study among German general practitioners (GPs), only 18% of GPs indicated that they frequently recommend apps to their patients (Wangler and Jansky, 2021). Another study with Australian HCPs found that even though the majority of HCPs were using health apps themselves, only half recommended apps to their patients (Byambasuren et al., 2019). Moreover, a study with Catalonian nurses identified that even though almost two-thirds of the nurses used apps regularly, 62% had never recommended an app while only 6.5% recommended apps frequently (Mayer et al., 2019).

While overall these findings indicate that HCPs do not commonly recommend apps to their patients, there are some notable differences between different types of apps. For example, a study with German HCPs found that among professionals who recommend apps, the most often recommended apps were the ones focused on prevention (e.g. maintaining a healthy lifestyle) or on self-management of health (e.g. monitoring blood pressure; Wangler and Jansky, 2021). Moreso, a majority of the physicians perceived apps for medication or appointment management (90%) as well as the management of the risk-symptoms (e.g. bodyweight; 88%) as beneficial. In contrast, a somehow smaller percentage of GPs were advocating for treating chronic diseases (59%). In the most recent study Wangler and Jansky (2023) found similar results, with a majority of the HCPs advocating for health apps for prevention, lifestyle change, and self-management of risk factors (e.g. weight), while a smaller proportion of HCPs advocated for monitoring and treating chronic diseases. Comparable findings were observed in a survey with Catalan nurses, who were found recommending apps for health promotion the most often while the least for monitoring of the patients (Mayer et al., 2019). These findings indicate that while overall recommendation of health apps may be low, improving recommendation of prevention oriented health apps may be easier than aiming to improve recommendation of more disease oriented apps as HCPs tend to consider apps such as symptom-tracking apps (Berkowitz et al., 2017) to be riskier.

Previous research has indicated that HCPs’ overall low readiness and willingness to recommend apps may be related to their inability to navigate their way through the jungle of the available applications and that they may lack a clear view of the quality and suitability of these apps for their specific group of patients. HCPs experience a range of barriers, such as not knowing where to look for reliable apps (Byambasuren et al., 2019), not having enough e-health related knowledge (Zakerabasali et al., 2021) and a lack of trust in app reliability (Wangler and Jansky, 2021; Zhang and Koch, 2015). The latter is not surprising as existing literature outlines the need for further work in areas of health app regulation and assessment (Essén et al., 2022). Overall, the lack of available guidance for judging app *quality* stands out as one of the critical barriers (Berkowitz et al., 2017; Dahlhausen et al., 2021). It is evident that if we want to promote the use of high quality apps as efficient and effective tools to support patients, clarity regarding the quality of different apps needs to be increased.

### Label for app quality assessment

**Figure 1.**
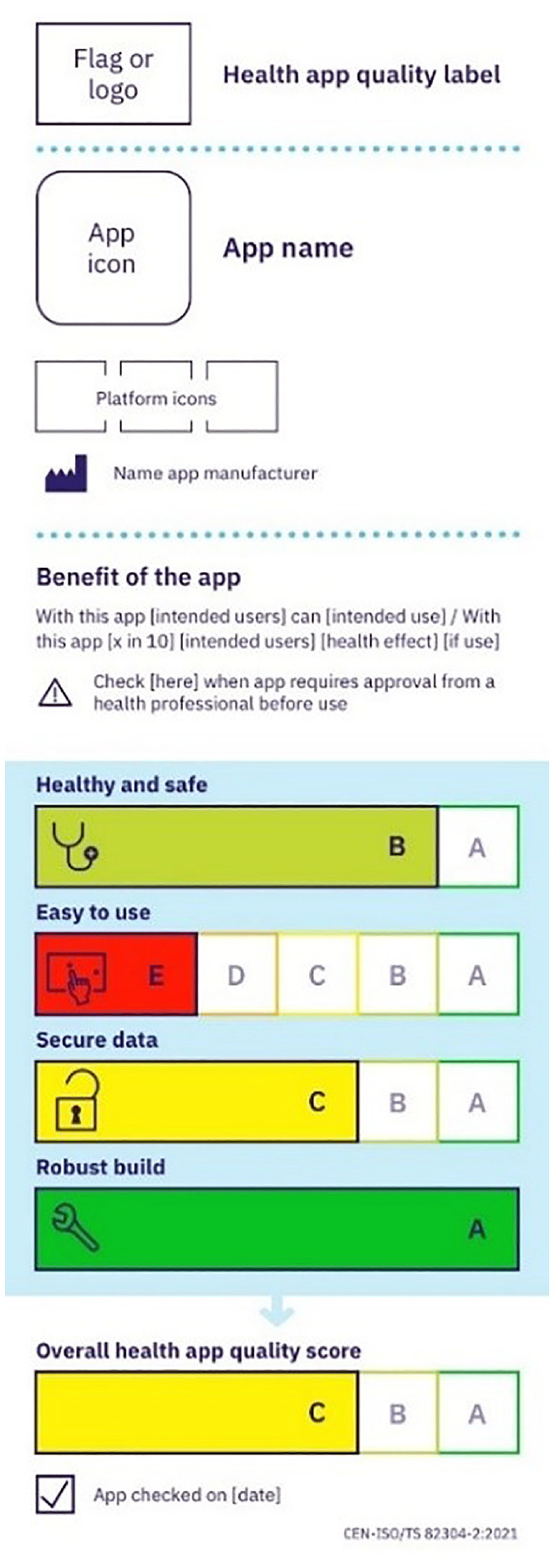
Quality label example.

To address this issue, recently, the International Organization for Standardization (ISO) published the Technical Specification (TS) 82304-2 Health and wellness apps—quality and reliability (ISO, 2021; Figure 1). This Technical Specification (further referred to as the quality label) provides a standardized app assessment framework and communication to provide clear, reliable, and accessible information about the quality of a particular health app. The quality label displays scores in four quality aspects, ranging from a green A (best score) to a red E (worst score). At the bottom, an overall weighted health app quality score (50% Healthy and safe, 15% Easy to use, 25% Secure data, and 10% Robust build) is provided.

The label and the related more detailed health app quality report is being developed to provide HCPs with the information they need to be able to make the decision whether a health and wellness app meets the relevant quality requirements to be recommended to a patient. However, since HCPs also experience other barriers to recommending health apps that may temper their willingness to recommend, such as negative outcome expectations regarding the effectiveness of apps in specific populations (Berkowitz et al., 2017), it is yet to be determined to what extent adding the quality label is indeed sufficient for promoting HCPs’ willingness to recommend apps. In particular, HCPs might perceive lower patient socioeconomic (SES) background as a risk factor and as a barrier to app use due to limitations in access or patients’ e-health affinity (e.g. Berkowitz et al., 2017) making them less willing to recommend health apps to these patients. The latter might have long-standing consequences as already pre-existing health inequalities for low SES patients (Phelan et al., 2010) might be further reinforced by the limitations on these patients’ opportunity to engage in digital health services (Helsper and Reisdorf, 2017). Besides, we know from other domains that presenting quality labels may only have limited effects on decision making (e.g. Siegrist et al., 2019; Vasiljevic et al., 2015), and that labeling effects might indeed differ based on individuals’ SES (Crockett et al., 2014) and therefore exacerbate existing health inequalities (Berkowitz et al., 2017; Song et al., 2021).

Hence, it is yet to be determined whether adding the health app quality label is indeed effective in increasing HCPs’ willingness to recommend apps (Aim 1), and whether its effect is similar for people with low versus high SES in order to not further increase the digital divide (Aim 2). Finally, considering previous findings indicating differences between different types of apps, it is worthwhile to explore whether adding the health app quality label enhances willingness to recommend all types of apps, or only the more prevention oriented types of apps that are generally considered less risky (Aim 3).

### The present study

To address our three aims, an experimental vignette study was designed in which HCPs are queried about their willingness to recommend high quality health apps across a range of situations. We manipulated whether or not the quality label was present in order to provide a first test of whether implementing the ISO 82304-2 quality label indeed increases HCPs’ willingness to recommend health apps. Right now, the quality label has been developed (ISO, 2021), but as a certification scheme is currently being developed it is yet to be implemented. While our findings will therefore necessarily be hypothetical in nature, gaining early insight on the potential effectiveness and whether or not effects may be moderated by SES of patients or types of apps, may yield important information to further shape and refine current and future implementation efforts. In addition to manipulating the presence of the quality label, we will manipulate two additional factors across the vignettes. First, we will describe different types of health apps within the vignettes to study the potential effect of the label across different types of health apps. For this purpose, based on the existing literature (e.g. Mustafa et al., 2022; Pires et al., 2020) we distinguished between three categories of health apps: (1) Prevention apps (i.e. prevention and behavior change-oriented apps), (2) Self-management apps (i.e. patient support such as management of a health issue-oriented apps), and (3) Healthcare apps (i.e. HCP support such as diagnostic and treatment-oriented apps). Secondly, we will manipulate whether the patient described in the vignette has a low or high SES background, to investigate if increases in HCPs’ willingness to recommend health apps will not solely benefit those with a high SES background and thus further enhance the digital divide (e.g. De Wilt et al., 2020).

We formulated three main hypotheses regarding the effect on our main dependent variable, willingness to recommend. Firstly, based on previous work which identified the lack of reliable information about the quality of health apps as one of the main barriers to app recommendation by HCPs (e.g. Byambasuren et al., 2020), we hypothesized that HCPs will be more likely to recommend health apps when the quality label is available (Aim 1). Secondly, based on previous findings (e.g. Berkowitz et al., 2017; Della Vecchia et al., 2022) we hypothesized that HCPs will be most willing to recommend prevention apps, followed by self-management apps, and the least willing to recommend healthcare apps, due to the latter’s highest potential risk of unwanted effects to the patient (Aim 2). Lastly, in line with previous research (e.g. De Wilt et al., 2020) we hypothesized that HCPs will be less likely to recommend health apps to patients who appear to be low SES than to patients from a high SES background (Aim 3). As there is a lack of previous literature on this topic, we did not formulate a priori hypotheses regarding any potential moderating effects of SES or type of app on the effectiveness of the quality label in improving willingness (Aims 2 and Aims 3) and consider these questions exploratory in nature.

As early testing of implementation outcomes is important to predict the appropriateness of interventions and direct subsequent implementation efforts, we also explored how HCPs scored the health app recommendation on the six APEASE criteria (Michie et al., 2014). APEASE stands for Acceptability, Practicability, Effectiveness, Affordability, Side effects, and Equity. These implementation criteria can be used at any stage of the intervention’s development to predict its suitability in the given context. In the present study, we will exploratively study how for each of the vignettes HCPs score on the APEASE criteria. This will allow us to compare the effect of the label on factors relevant to eventual uptake by HCPs other than willingness, and it will help us to get a richer view on the potential of the label to promote recommendations of health apps by HCPs.

## Method

### Participants

Participants in the study were HCPs, including physicians, nurses, or any other practicing HCPs (e.g. pharmacists and physiotherapists). To be able to take part in the study, HCPs had to be 18 years of age or older, be resident and licensed to practice in the EU, a European Free Trade Association (EFTA) country, or Ukraine, and be able to comprehend, read, and write in English.

Recruitment lasted ten weeks and in total 118 HCPs completed the questionnaire. Out of these, two participants were removed due to not meeting the inclusion criteria (not from EU/EFTA country or Ukraine). Hence, the final sample consisted of 116 participants (58.6% female (*n* = 68), *M*_age_ = 41.70, SD_age_ = 12.21). On average, HCPs were practicing for 15 years (SD = 11.24) and the majority of HCPs were physicians (56.9%). Out of the physicians, the majority were family medicine physicians (40.9%), followed by physicians with a variety of different specializations, from anesthesiology to urology. Most HCPs were practicing in an urban area (82.8%). Lastly, the majority of the HCPs were practicing in the hospital inpatient setting (25.9%) or primary healthcare practice (24.1%). Most HCPs were residing in the Netherlands (13.8%), Croatia and Lithuania (12.9% each), Greece (12.1%), and Germany (10.3%).

### Design

The study consisted of an online experimental vignette study and used a 2 Label (absent vs present; between subjects) × 3 Type of App (prevention vs self-monitoring vs healthcare; within subjects) × 2 SES (low vs high; within subjects) mixed design. This resulted in a total of 12 vignettes, of which each participant thus saw six vignettes (either all with or all without the label). The main dependent variable was HCPs’ willingness to recommend health apps. One item addressing each of the six APEASE criteria was then administered to assess the effect of the label on the acceptability, practicability, effectiveness, affordability, side effects, and equity of recommending health apps (Michie et al., 2014).

### Procedure

Participant recruitment took place online, during the first quarter of 2023. After providing informed consent online by ticking a box, participants were randomly distributed over the two groups. Participants in the quality label-present group first read about the meaning and purpose of the quality label and saw three examples of the label (Supplemental File 1). Participants in the quality label-absent group did not receive this information and instead started with the first vignette.

The research protocol was assessed and approved by the Science Committee of the Public Health and Primary Care Department (PHEG) at Leiden University Medical Centre’s (LUMC; nr WSC-2022-34/ PvN).

### Materials

#### Vignettes

We carefully constructed twelve text-based scenarios. All vignettes followed the same structure and described a situation in which the HCP would see a particular patient. For a complete overview of all materials and instructions, see Supplemental Material 1.

#### Outcome measures

Each vignette was followed by nine questions. First, two questions assessed whether participants recognized the type of patient from the description and whether they typically recommend the type of app described in the vignette. Then, the main outcome measure was assessed by one item (*How willing are you to recommend these types of health apps to this type of patient?*) on a 7-point scale ranging from 1 (Very unwilling) to 7 (Very willing). The remaining six items were constructed based on the APEASE framework and were aimed to further investigate HCPs’ willingness to recommend health apps to patients, measured on 7-point Likert scale.

Participants were also presented with seven items to assess their overall e-health affinity and eight questions concerning their demographic characteristics.

#### Data analyses

To assess the effect of the label on willingness to recommend and on whether any effects are moderated by app type or patients’ background, a mixed ANOVA was conducted with willingness to recommend the app as the dependent variable and the Label (absent vs present) as between-subjects factor and Type of App (prevention vs self-management vs healthcare) and SES (low vs high) as within-subject factors. Additionally, six separate repeated measures ANOVAs were performed with each of the APEASE criteria as dependent variables.

The hypotheses, experimental design, and basic analysis pipeline were preregistered at AsPredicted (#118530). We were not able to reach the preregistered number of participants.

## Results

### Data processing

*Z* scores were calculated to detect outliers (3SDs above or below the mean) for the main outcome of willingness to recommend high quality health apps to patients. Analyses were performed with and without the exclusion of outliers. As this did not affect the results only the findings based on the full sample will be reported.

### Descriptives and intercorrelations

Overall, the majority of HCPs had previously recommended health apps to patients at least once (70.7%). Out of these, the majority had recommended self-management apps (65.8%), followed by prevention (51.2%) and healthcare apps (29.3%). Within this subset of participants, the majority indicated to be recommending apps less than once per month (47.6%) or on a monthly basis (32.9%). Most HCPs indicated that patients either never ask them for health apps (44%) or ask for app recommendations less than once per month (33.6%). Within the subsample of HCPs who had received requests for recommendations, most received requests for self-management apps (60%), followed by prevention (47.7%) and healthcare apps (36.9%).

Overall, HCPs reported relatively high willingness to recommend apps (*M* = 5.08, SD = 1.27). This was also reflected in relatively high scores for acceptability (*M* = 4.93, SD = 1.22), practicability (*M* = 4.53, SD = 1.28), effectiveness (*M* = 4.21, SD = 1.11), and affordability (*M* = 4.88, SD = 1.10). Furthermore, moderate repeated measures correlations were found between willingness on the one hand and acceptability (*r* = 0.62, *p* = 0.002), practicability (*r* = 0.54, *p* = 0.003), effectiveness (*r* = 0.57, *p* < 0.001), and affordability (*r* = 0.34, *p* = 0.001) on the other hand. Full descriptive statistics and repeated measures correlations^1^ for willingness and the APEASE criteria are presented in Table 1.

**Table 1.** *M*, SD, and repeated measures correlations for willingness and the APEASE criteria (*N* = 116).

	*M* (*SD*)	1	2	3	4	5	6	Willingness
1. Acceptability	4.93 (1.22)	—						
2. Practicability	4.53 (1.28)	.60***	—					
3. Effectiveness	4.21 (1.11)	.61***	.70***	—				
4. Affordability	4.88 (1.10)	.45***	.56***	.49***	—			
5. Side-effects	2.70 (1.40)	−.06	.03	.00	.06	—		
6. Equity	3.76 (1.23)	.16***	.21***	.14**	.17***	.08	—	
Willingness	5.08 (1.27)	.62***	.54***	.57***	.34***	.01	.12**	—

* *p* < .05. **p < .01. ***p < .001.

### Randomization check

Independent samples *t*-tests (for continuous demographic variables: age and years of practice) and Chi-squared analyses (for categorical variables: gender, country, location, occupation, practice setting, and whether HCPs previously recommended or were asked by patients to recommend health apps) were conducted to check for unintended differences between the two groups (Label-absent vs Label-present). No differences between the two groups were identified (all *p*’s > 0.100).

### Main analyses

#### Willingness to recommend health apps

A mixed ANOVA was conducted with the Label (absent vs present) as between-subjects factor, Type of App (prevention vs self-management vs healthcare), and SES (low vs high) as within-subject variables, and willingness to recommend the health app as the dependent variable. A significant main effect of the Label was identified, *F*(1, 114) = 8.01 *p* = 0.006, η_p_^2^ = 0.07. As hypothesized, HCPs in the quality label-present group (*M* = 5.46, SD = 1.23) were more willing to recommend health apps to patients than HCPs in the label-absent group (*M* = 4.81, SD = 1.23). The main effect of Type of App was also significant, *F*(1.87, 213.69) = 7.87, *p* < 0.001, η_p_^2^ = 0.06. However, contrary to the second hypothesis, a Bonferroni post-hoc analysis to disentangle this main effect showed that willingness was significantly higher for self-management apps (*M* = 5.42, SD = 1.36) than for prevention apps (*M* = 5.04, SD = 1.50), *p* = 0.004, 95% CI [0.10, 0.66], or healthcare apps (*M* = 4.94, SD = 1.56), *p* < 0.001, 95% CI [0.18, 0.77]. No difference in willingness was found between prevention and healthcare apps (*p* = 1). Finally, in line with the third hypothesis, the main effect of SES was also significant, *F*(1, 114) = 28.09, *p* < 0.001, η_p_^2^ = 0.20. HCPs were more willing to recommend apps to patients with a high (*M* = 5.38, SD = 1.31) than a low SES background (*M* = 4.89, SD = 1.38).

The effect of the quality label on willingness to recommend high quality health apps was not moderated by the Type of App or SES of the patient (all *p*’s > 0.718). Similarly, no three-way interaction (Label × Type of App × SES) was found (*p* = 0.211). This indicates that the beneficial effect of the label on willingness to recommend is similar for high versus low SES patients and for the three types of apps. There was, however, a significant interaction between the Type of App and SES, *F*(2, 228) = 4.45, *p* = 0.013, η_p_^2^ = 0.04. Simple main effects revealed that within low SES participants, there was a significant difference in willingness between self-management (*M* = 5.23, SD = 1.59 ) and prevention apps (*M* = 4.66, SD = 1.77), *p* = 0.003, and self-management and healthcare apps (*M* = 4.76, SD = 1.81), *p* = 0.01, but not between prevention and healthcare apps, *p* = 1. In turn, within high SES participants, none of the apps differed significantly from each other (all *p*’s > 0.140 ), except for self-management (*M* = 5.61, SD = 1.44) and healthcare apps (*M* = 5.12, SD = 1.66), *p* < 0.001 (further details in Table 3 in the Supplemental File 2).

#### APEASE criteria

Similar 2 × 2 × 3 mixed ANOVAs were conducted with each of the six APEASE criteria separately as the dependent variable.

Regarding our main effect of interest, the effect of the Label, a significant difference was found for acceptability, *F*(1, 114) = 6.97, *p* = 0.009, η_p_^2^ = 0.06. That is, HCPs in the quality label-present group were more acceptant of recommending high quality health apps to patients (*M* = 5.27, SD = 1.19) than HCPs in the label-absent group (*M* = 4.68, SD = 1.19). For all other APEASE-related outcomes, there was no effect of the label, meaning that the HCPs in the two groups did not differ on criteria of practicability, effectiveness, affordability, side-effects, and equity in recommending high quality health apps to their patients (see Table 2).

**Table 2.** Mixed ANOVAs for the effect of Label on the APEASE criteria.

Criteria	*M* (*SD*)		*F*(1, 114)	*p*	η_p_^2^
	Label-absent group	Label-present group			
Acceptability	4.68 (1.19)	5.27 (1.19)	6.97	.009	.06
Practicability	4.37 (1.27)	4.77 (1.27)	2.85	.094	.02
Effectiveness	4.15 (1.11)	4.29 (1.11)	.42	.519	.00
Affordability	4.78 (1.10)	5.02 (1.09)	1.34	.249	.01
Side effects	2.67 (1.41)	2.74 (1.41)	.07	.792	.00
Equity	3.81 (1.23)	3.69 (1.24)	.294	.588	.00

For Type of App, significant main effects were found for four out of six APEASE criteria: acceptability, practicability, effectiveness, and side effects (all *p*’s < 0.003). For acceptability, practicability, and effectiveness, scores were highest for self-management apps. For side effects, the means for prevention apps were significantly lower than for healthcare apps (for a full overview, see Table 1 in the Supplemental Material 2). A significant main effect of SES was found on all the APEASE criteria (*p*’s < 0.001), except on side-effects (*p* = 0.957). The direction of these effects was similar for all five significant criteria, with more favorable scores for high SES patients (full details in Table 2 in the Supplemental Material 2).

The effect of the quality label on the APEASE criteria was not moderated by the Type of App or SES (all *p*’s > 0.09). Similarly, no three-way interaction (Label × Type of App × SES) was found (all *p*’s > 0.146). However, significant interaction effects of Type of App and SES were found on acceptability (*F*(1.89, 215.55) = 3.15, *p* = 0.048, η_p_^2^ = 0.03), practicability (*F*(2, 228) = 4.91, *p* = 0.008, η_p_^2^ = 0.04), effectiveness (*F*(1.89, 215.86) = 9.63, *p* < 0.001, η_p_^2^ = 0.08), and affordability (*F*(1.86, 211.96) = 5.74, *p* = .005, η_p_^2^ = 0.05; for details, please refer to Tables 4–7 in the Supplemental Material 2).

## Discussion

With this study, we aimed to evaluate HCPs’ willingness to recommend high quality health apps with and without the ISO/TS 82304-2 health app quality label. Results showed that HCPs were more willing to recommend health apps when they were provided with the quality label as compared to the situation where the label was not added. While we did find main effects of type of app and SES, crucially, the effect of adding the label on willingness to recommend was not affected by type of app or patients’ SES indicating that the positive effect of the quality label did not solely benefit certain types of apps or patients, but that the effect occurred regardless of type of app or patient’s SES. Lastly, exploration of the APEASE criteria indicated that overall, the acceptability, practicability, perceived effectiveness and affordability of recommending health apps was already scored relatively high, as reflected in the 70% of respondents who had recommended a health app at least once, but that acceptability increased even further when apps were accompanied by the quality label. This criterion was also most strongly correlated to willingness to recommend, indicating that this may potentially be an important driving force behind their higher willingness to recommend apps when the label is included.

This study thus provides a first indication that the quality label increases willingness of HCPs to recommend health apps to their patients (Aim 1). Previous literature had identified several barriers for app recommendation, including not knowing where to find reliable apps (Byambasuren et al., 2019) and concerns about app security (Gagnon et al., 2016) and quality (Wangler and Jansky, 2021). Providing HCPs with the quality label intends to address such barriers and our study reveals that this may successfully increase willingness to recommend health apps. Given the medium effect size, this endeavor to use the quality label to establish healthcare requirements for health and wellness apps (ISO, 2021) may potentially change clinical practice toward higher adoption of e-health. In turn, as the quality label was inspired by other existing labels (e.g. the EU Energy label), findings contribute to existing evidence of acceptability and the potential of such labels in guiding individuals in making well-informed choices. Yet, it is important to note that while willingness is an important factor for behavior change, more may be needed to put HCPs’ intentions into action (i.e. intention-behavior gap; Webb and Sheeran, 2006).

Importantly, our study suggests that this beneficial effect of the label was not affected by the type of app (Aim 2). Previous studies had identified that HCPs see potential for recommending prevention or self-monitoring apps (Wangler and Jansky, 2021), but are concerned about unwanted effects and cases in which symptom-tracking apps are used to report worsening or critical symptoms that would require immediate HCP attention (Berkowitz et al., 2017). This study found similar differences between types of apps, with HCPs being most willing to recommend self-management apps. Interestingly, however, the label seems to increase willingness across all three types of apps, thereby reassuring HCPs with sufficient information regarding app quality regardless of the type of app. Given the rapid increase in the number of apps available across all types of apps (Iqvia Institute, 2021), this implies that the label can effectively guide HCPs through the ever-increasing jungle of existing apps. Once developed, the related and more detailed health app quality report may further increase HCPs willingness to recommend high quality apps.

Another important finding of this study is that the beneficial effect of the label is not affected by the patients’ SES (Aim 3). That is, even though in general HCPs remain slightly more reluctant to recommend apps to lower SES patients (c.f., Bernheim et al., 2008; Dawson et al., 2022), the label does not further increase the digital divide because of differential effects of the label on the likelihood of apps recommendations by the HCP. At the same time, our findings also imply that the label may not decrease inequalities, but given concerns about intervention-generated inequalities (Veinot et al., 2018) this is a promising effect by itself. This finding does not rule out potential differences in adoption of apps by patients of different SES backgrounds, but at least the label ensures a higher likelihood of HCPs recommending health apps. Given that patients may feel more motivated to use apps that are recommended to them by their HCPs (Jezrawi et al., 2022; Lupiáñez-Villanueva et al., 2020), this is relevant to stimulate health app usage regardless of SES backgrounds.

### Limitations and future research

Even though this study provides first evidence for the beneficial effect of the label on HCPs’ willingness to recommend health apps to their patients, several limitations deserve to be mentioned. First and foremost, this study used hypothetical scenarios, which is not uncommon in clinical decision-making research with HCPs (e.g. Evans et al., 2015; Wilson et al., 2017). We carefully constructed these scenarios in collaboration with HCPs to ensure high ecological validity, but these hypothetical situations cannot fully account for the complexity of real-world (clinical) decision making. Relatedly, the main dependent variable (willingness to recommend health apps) does not equal actual behavior (change). As alluded to before, extensive research has identified the gap between intentions and behavior (i.e. intention-behavior gap; Webb and Sheeran, 2006). Therefore, it remains to be seen whether our promising results will translate into actual changes in clinician behavior. Yet, studying implementation factors early on is necessary for successful implementation of innovations (Damschroder et al., 2022) making the use of hypothetical situations with willingness as main dependent variable an inevitable and important step.

Another limitation relates to our sample size and sampling strategy. We aimed to recruit a large and diverse sample of HCPs across Europe. HCPs are known as a difficult-to-recruit population (e.g. Cave et al., 2009) and therefore we made use of various partner organizations with significant memberships. Our study, however, turned out to be no exception despite our collaborations, perhaps in part due to workloads, post-COVID-19, and otherwise (Doleman et al., 2023; Fernández-Aguilar et al., 2021). Still, and especially given the within-subjects factors in the study design, we were able to address the main aims with sufficient statistical power. Finally, by using an online study to investigate willingness to recommend health apps to patients, we may have run the risk of selection bias where HCPs who were more familiar with e-health were more eager to participate. While we cannot exclude this possibility, which given adoption rates of health app prescription behavior in Germany seems likely (Dahlhausen et al., 2021; Richter et al., 2022), we did put effort in using many different options for participant recruitment.

As a next step in studying the potential impact of the label, future research should report its impact on actual recommendation behavior of HCPs. Based on our findings and in line with existing research evidence (e.g. Curtis et al., 2017), tools such as the APEASE criteria (Michie et al., 2014) can be successfully used for gaining insights into implementation criteria for HCPs’ recommendation behavior. Moreover, while the label does seem to increase willingness to recommend high quality health apps, it likely does not address all relevant barriers that are experienced in daily practice. Further determinants of HCPs’ behavior, like self-efficacy (Bandura and Walters, 1977) or outcome expectancies (Bandura, 1986, 2001), may still require attention and further Behavior Change Techniques (Michie et al., 2013) may be needed to address those determinants successfully. Moreover, given recent insights into the role of automatic processes in HCPs’ behavior (Potthoff et al., 2019), future research should also seek to understand how implementing the label can successfully align with daily routines in practice (Potthoff et al., 2022). Finally, an important step for future research would be to find suitable techniques to change HCPs’ behavior in such a way that it aids in decreasing the digital divide and health inequalities. High scores of apps in the quality aspect “Easy to use,” reflected on the label, may contribute.

## Conclusion

This study set out to experimentally test whether the ISO/TS 82304-2 health app quality label increased HCPs’ willingness to recommend apps, and whether this hypothesized increase would be moderated by the type of app or patients’ SES. Results were promising as this study provided the first preliminary evidence of the label’s effectiveness in increasing HCPs’ willingness to recommend health apps. Importantly, the positive effect of the label did not only occur for specific types of apps or high SES patients but was robust for the three types of apps (prevention, self-management, and healthcare) and across the patient groups (low vs high SES).

## Supplemental Material

sj-docx-1-hpq-10.1177_13591053241258205 – Supplemental material for Value of a quality label and European healthcare professionals’ willingness to recommend health apps: An experimental vignette study Supplemental material, sj-docx-1-hpq-10.1177_13591053241258205 for Value of a quality label and European healthcare professionals’ willingness to recommend health apps: An experimental vignette study by Ieva Biliunaite, Laurens van Gestel, Petra Hoogendoorn and Marieke Adriaanse in Journal of Health Psychology

sj-docx-2-hpq-10.1177_13591053241258205 – Supplemental material for Value of a quality label and European healthcare professionals’ willingness to recommend health apps: An experimental vignette study Supplemental material, sj-docx-2-hpq-10.1177_13591053241258205 for Value of a quality label and European healthcare professionals’ willingness to recommend health apps: An experimental vignette study by Ieva Biliunaite, Laurens van Gestel, Petra Hoogendoorn and Marieke Adriaanse in Journal of Health Psychology

## Notes

1. Due to the repeated measures structure of the data, we decided to report on repeated measures correlations instead of regular correlations. See Bakdash JZ and Marusich LR (2017).
