# Supplementary material for: Value of a quality label and European healthcare professionals’ willingness to recommend health apps: An experimental vignette study
Source: J Health Psychol. 2024 Aug 2;30(6):1278–90. doi: 10.1177/13591053241258205 (PMC12052922; doi:10.1177/13591053241258205)
Supplement: sj-docx-1-hpq-10.1177_13591053241258205 – Supplemental material for Value of a quality label and European healthcare professionals’ willingness to recommend health apps: An experimental vignette study [file sj-docx-1-hpq-10.1177_13591053241258205.docx]

**Supplementary file 1**

## A-Instructions quality label-present group

Thank you for agreeing to take part in this survey for European healthcare professionals.

In July of 2021 the International Organization for Standardization (ISO) published Technical Specification (TS) 82304-2 ‘health and wellness apps – quality and reliability’. Core content of this TS is a health app quality assessment framework and a health app quality label.

The health app quality label, inspired by the EU Energy label, indicates the quality of a health app in four quality aspects and an overall health app quality score. All scores can range from a green A (best score) to a red E (worst score). *Healthy and safe* accounts for 50% in the overall score, *Secure data* for 25%, *Easy to use* for 15%, and *Robust build* for 10%.

These are some examples of the label:

Example 1: Example 2: Example 3:


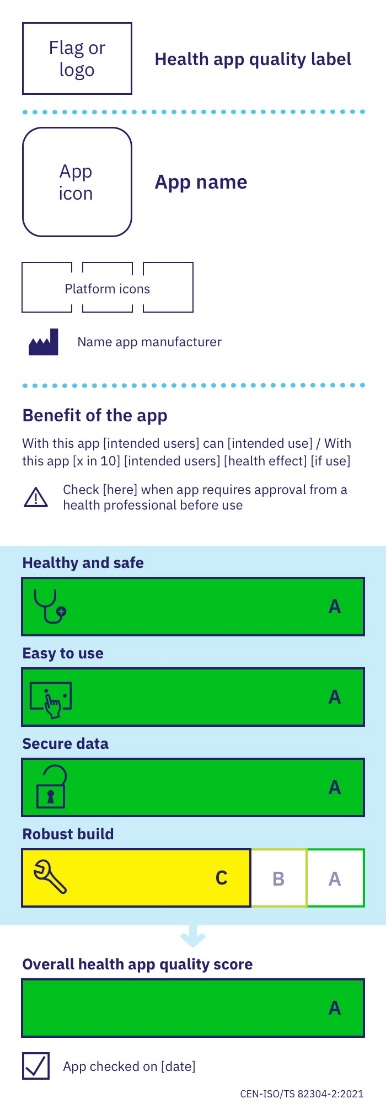

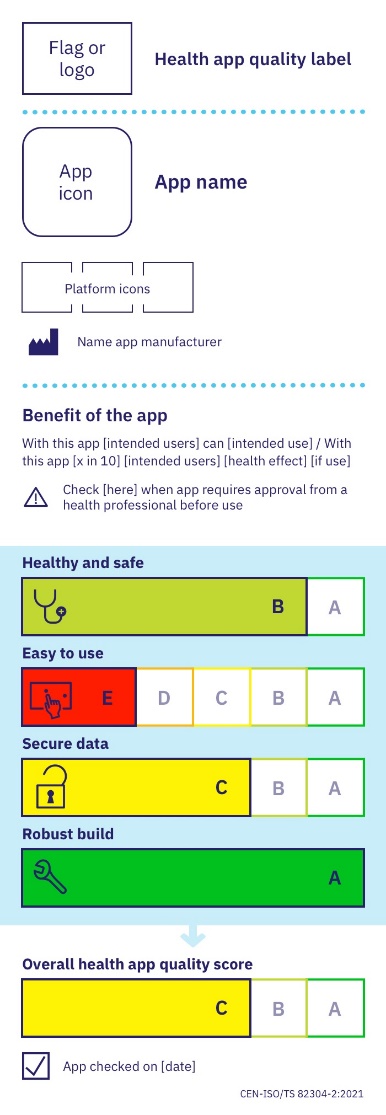

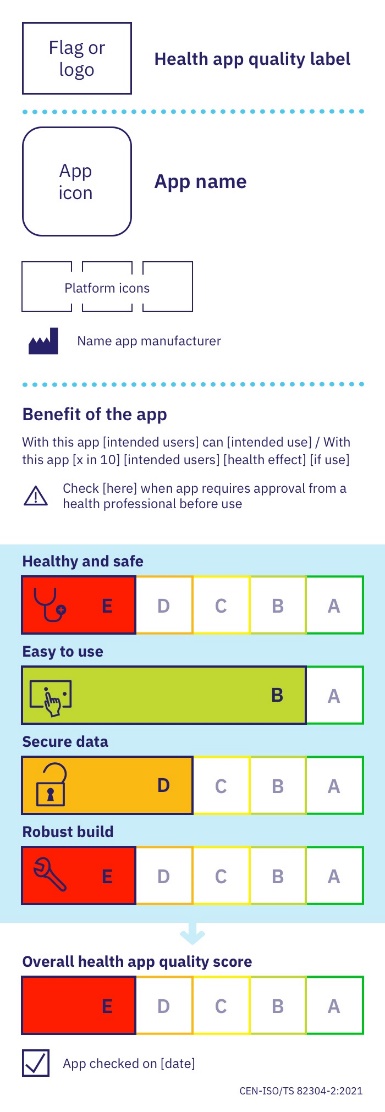


To qualify for a label an app manufacturer has to provide evidence. Accredited app assessors evaluate the evidence provided. In their assessment they use an ISO 17065 handbook for app assessment (the so called certification scheme). This handbook is aligned with EU values and EU legislation (Medical Device Regulation, General Data Protection Regulation, etc.) and evaluated as proportional / adequate by healthcare professionals.

The quality label is accompanied by a more detailed health app quality report. This report reveals for which quality requirements evidence proved sufficient and further details. Intent of the label and report is to give healthcare professionals the information they need on the quality of a health app.

Below, we will present six short cases of different health situations, patients and types of health apps. Please read them carefully. Following each of the six cases, we will ask you a set of short questions.

## B-List of short stories (vignettes) for quality label-present group

SHORT STORY 1

Please imagine seeing a patient in your role as a healthcare professional. This patient has what is considered a low socioeconomic background, which means that the patient is relatively low educated and/or has an income that is generally considered low. The patient does not have a chronic illness yet, but due to unhealthy behavior is at risk for developing a chronic condition, such as obesity or Chronic Obstructive Pulmonary Disease (COPD). For this type of patient, health apps are available to help in taking preventive measures such as a healthier diet, exercising regularly, and, in the case of COPD, reducing or quitting smoking. For example, these apps can help a patient track their exercise routine and calorie intake or provide additional tips for a healthy lifestyle.

The quality label that you have seen at the start of the survey is readily available for checking the quality of these apps.

SHORT STORY 2

Please imagine seeing a patient in your role as a healthcare professional. This patient has what is considered a high socioeconomic background, which means that the patient is relatively high educated and/or has an income that is generally considered high. The patient does not have a chronic illness yet, but due to unhealthy behavior is at risk for developing a chronic condition, such as obesity or Chronic Obstructive Pulmonary Disease (COPD). For this type of patient, health apps are available to help in taking preventive measures such as a healthier diet, exercising regularly, and, in the case of COPD, reducing or quitting smoking. For example, these apps can help a patient track their exercise routine and calorie intake or provide additional tips for a healthy lifestyle.

The quality label that you have seen at the start of the survey is readily available for checking the quality of these apps.

SHORT STORY 3

Please imagine seeing a patient in your role as a healthcare professional. This patient has what is considered a low socioeconomic background, which means that the patient is relatively low educated and/or has an income that is generally considered low. The patient has been diagnosed with a chronic illness such as type 2 diabetes or heart failure. For this type of patient, health apps are available to help in monitoring their symptoms, such as fatigue or shortness of breath with activity, and in their self-management, such as adhering to dietary guidelines. For example, these apps can help **a patient to regularly monitor his/hers blood pressure, heart rate, or glucose levels as well as to help in tracking adherence to medication, food, or calorie intake.**

The quality label that you have seen at the start of the survey is readily available for checking the quality of these apps.

SHORT STORY 4

Please imagine seeing a patient in your role as a healthcare professional. This patient has what is considered a high socioeconomic background, which means that the patient is relatively high educated and/or has an income that is generally considered high. The patient has been diagnosed with a chronic illness such as type 2 diabetes or heart failure. For this type of patient, health apps are available to help in monitoring their symptoms, such as fatigue or shortness of breath with activity, and in their self-management, such as adhering to dietary guidelines. For example, these apps can help **a patient to regularly monitor his/hers blood pressure, heart rate, or glucose levels as well as to help in tracking adherence to medication, food, or calorie intake.**

The quality label that you have seen at the start of the survey is readily available for checking the quality of these apps.

SHORT STORY 5

Please imagine seeing a patient in your role as a healthcare professional. This patient has what is considered a low socioeconomic background, which means that the patient is relatively low educated and/or has an income that is generally considered low. The patient has been diagnosed with a chronic illness such as chronic pain or depression. For this type of patient, health apps are available to provide medical intervention and directly address their symptoms, such as insomnia, anxiety, and fatigue. For example, these apps can help a patient by providing Cognitive Behaviour Therapy (CBT) to address anxiety or depression, or, by providing a focused fitness program to reduce chronic back pain.

The quality label that you have seen at the start of the survey is readily available for checking the quality of these apps.

SHORT STORY 6

Please imagine seeing a patient in your role as a healthcare professional. This patient has what is considered a high socioeconomic background, which means that the patient is relatively high educated and/or has an income that is generally considered high. The patient has been diagnosed with a chronic illness such as chronic pain or depression. For this type of patient, health apps are available to provide medical intervention and directly address their symptoms, such as insomnia, anxiety, and fatigue. For example, these apps can help a patient by providing Cognitive Behaviour Therapy (CBT) to address anxiety or depression, or, by providing a focused fitness program to reduce chronic back pain.

The quality label that you have seen at the start of the survey is readily available for checking the quality of these apps.

## C-List of short stories (vignettes) for quality label-absent group

SHORT STORY 1

Please imagine seeing a patient in your role as a healthcare professional. This patient has what is considered a low socioeconomic background, which means that the patient is relatively low educated and/or has an income that is generally considered low. The patient does not have a chronic illness yet, but due to unhealthy behavior is at risk for developing a chronic condition, such as obesity or Chronic Obstructive Pulmonary Disease (COPD). For this type of patient, health apps are available to help in taking preventive measures such as a healthier diet, exercising regularly, and, in the case of COPD, reducing or quitting smoking. For example, these apps can help a patient track their exercise routine and calorie intake or provide additional tips for a healthy lifestyle.

SHORT STORY 2

Please imagine seeing a patient in your role as a healthcare professional. This patient has what is considered a high socioeconomic background, which means that the patient is relatively high educated and/or has an income that is generally considered high. The patient does not have a chronic illness yet, but due to unhealthy behavior is at risk for developing a chronic condition, such as obesity or Chronic Obstructive Pulmonary Disease (COPD). For this type of patient, health apps are available to help in taking preventive measures such as a healthier diet, exercising regularly, and, in the case of COPD, reducing or quitting smoking. For example, these apps can help a patient track their exercise routine and calorie intake or provide additional tips for a healthy lifestyle.

SHORT STORY 3

Please imagine seeing a patient in your role as a healthcare professional. This patient has what is considered a low socioeconomic background, which means that the patient is relatively low educated and/or has an income that is generally considered low. The patient has been diagnosed with a chronic illness such as type 2 diabetes or heart failure. For this type of patient, health apps are available to help in monitoring their symptoms, such as fatigue or shortness of breath with activity, and in their self-management, such as adhering to dietary guidelines. For example, these apps can help **a patient to regularly monitor his/hers blood pressure, heart rate, or glucose levels as well as to help in tracking adherence to medication, food, or calorie intake.**

SHORT STORY 4

Please imagine seeing a patient in your role as a healthcare professional. This patient has what is considered a high socioeconomic background, which means that the patient is relatively high educated and/or has an income that is generally considered high. The patient has been diagnosed with a chronic illness such as type 2 diabetes or heart failure. For this type of patient, health apps are available to help in monitoring their symptoms, such as fatigue or shortness of breath with activity, and in their self-management, such as adhering to dietary guidelines. For example, these apps can help **a patient to regularly monitor his/hers blood pressure, heart rate, or glucose levels as well as to help in tracking adherence to medication, food, or calorie intake.**

SHORT STORY 5

Please imagine seeing a patient in your role as a healthcare professional. This patient has what is considered a low socioeconomic background, which means that the patient is relatively low educated and/or has an income that is generally considered low. The patient has been diagnosed with a chronic illness such as chronic pain or depression. For this type of patient, health apps are available to provide medical intervention and directly address their symptoms, such as insomnia, anxiety, and fatigue. For example, these apps can help a patient by providing Cognitive Behaviour Therapy (CBT) to address anxiety or depression, or, by providing a focused fitness program to reduce chronic back pain.

SHORT STORY 6

Please imagine seeing a patient in your role as a healthcare professional. This patient has what is considered a high socioeconomic background, which means that the patient is relatively high educated and/or has an income that is generally considered high. The patient has been diagnosed with a chronic illness such as chronic pain or depression. For this type of patient, health apps are available to provide medical intervention and directly address their symptoms, such as insomnia, anxiety, and fatigue. For example, these apps can help a patient by providing Cognitive Behaviour Therapy (CBT) to address anxiety or depression, or, by providing a focused fitness program to reduce chronic back pain.

## D-List of questions after every vignette

1) How often do you see this type of patient?

Very frequently

1 2 3 4 5 6 7

Never

2) How often do^[[1]](#footnote-1)^ you recommend these types of health apps to this type of patient?

Very frequently

1 2 3 4 5 6 7

Never

3) How willing are you to recommend these types of health apps to this type of patient?

1 2 3 4 5 6 7

Very willing

Very unwilling

4) How acceptable is recommending these types of health apps to this type of patient?

Very acceptable

1 2 3 4 5 6 7

Very unacceptable

5) How practical is recommending these types of health apps to this type of patient?

Very practical

1 2 3 4 5 6 7

Very unpractical

6) How effective is recommending these types of health apps to this type of patient?

Very effective

1 2 3 4 5 6 7

Very ineffective

7) How affordable are these types of health apps to this type of patient?

Very affordable

1 2 3 4 5 6 7

Very unaffordable

8) How likely is this type of patient to experience side-effects due to the use of these types of health apps

Very likely

1 2 3 4 5 6 7

Very unlikely

9)To what extent does recommending these types of health apps to this type of patient have an effect on health inequalities?

Would increase health inequalities very much

Would decrease

1 2 3 4 5 6 7

health inequalities

very much

1. For quality label-present group this item was formulated using ‘’would’’ instead of ‘’do’’ [↑](#footnote-ref-1)
