# Supplementary material for: Value of a quality label and European healthcare professionals’ willingness to recommend health apps: An experimental vignette study
Source: J Health Psychol. 2024 Aug 2;30(6):1278–90. doi: 10.1177/13591053241258205 (PMC12052922; doi:10.1177/13591053241258205)
Supplement: sj-docx-2-hpq-10.1177_13591053241258205 – Supplemental material for Value of a quality label and European healthcare professionals’ willingness to recommend health apps: An experimental vignette study [file sj-docx-2-hpq-10.1177_13591053241258205.docx]

**Supplementary file 2**

**APEASE & Type of App**

Results of the 2x2x3 mixed ANOVAs for Type of App with each of the six of the APEASE criteria as dependent variables are presented in **Table 1** Bonferroni correction was performed via SPSS to adjust for multiple testing. The results of the Bonferroni post hoc test are presented below the table.

**Table 1**. Mixed ANOVAs for the main effect of Type of App on the APEASE criteria

| Criteria | *M(SD)* | | | *F* (df) | *p* | *η_p_^2^* |
| --- | --- | --- | --- | --- | --- | --- |
|  | Prevention apps | Self-management apps | Healthcare apps |  |  |  |
| Acceptability | 4.92 (1.42) | 5.34 (1.41) | 4.67 (1.40) | 16.79  (1.80, 205.25 | <.001 | .13 |
| Practicability | 4.46 (1.50) | 4.83 (1.40) | 4.41 (1.42) | 9.77  (2, 228) | <.001 | .08 |
| Effectiveness | 4.01 (1.33) | 4.59 (1.29) | 4.07 (1.36) | 15.78  (1.89, 215.89) | <.001 | .12 |
| Affordability | 4.89 (1.24) | 4.97 (1.28) | 4.83 (1.18) | 1.44  (2, 228) | .239 | .01 |
| Side effects | 2.52 (1.50) | 2.75 (1.59) | 2.85 (1.54) | 6.06  (1.87, 213.75) | .003 | .05 |
| Equity | 3.85 (1.32) | 3.71 (1.46) | 3.69 (1.32) | 1.76  (2, 228) | .175 | .01 |

Bonferroni post hoc results are as follows:

**Acceptability**: results indicated acceptability to be significantly higher for self-management apps than for prevention apps (*p* < .001, 95% *CI* = [ .18, .65]), or healthcare apps (*p* < .001, 95% *CI* = [ .38, .97]). No difference in acceptability was found between prevention and healthcare apps *(p* = .150).

**Practicability**: results indicated practicability to be significantly higher for self-management apps than for prevention apps (*p* < .001, 95% *CI* = [ .13, .61]), or healthcare apps (*p* < .001, 95% *CI* = [ .18, .67]). No difference in practicability was found between prevention and healthcare apps *(p* = 1).

**Effectiveness**: results indicated effectiveness to be significantly higher for self-management apps than for prevention apps (*p* < .001, 95% *CI* = [ .33, .83]), or healthcare apps (*p* < .001, 95% *CI* = [ .24, .79]). No difference in effectiveness was found between prevention and healthcare apps *(p* = 1).

**Side-effects**: results indicated side-effects to be significantly lower for prevention apps as compared to self-management (*p* = .009, 95% *CI* = [ -.59, -.07]) or healthcare apps (*p* = .050, 95% *CI* = [ -.46, .00]). The differences for healthcare and self-management apps was not significant *(p* = .748).

**APEASE & SES**

Results of the 2x2x3 mixed ANOVAs for SES with each of the six of the APEASE as dependent variables are presented in Table 2.

**Table 2.** Mixed ANOVAs for the effect of SES on the APEASE criteria

| Criteria | *M(SD)* | | *F* (1,114) | *p* | *η_p_^2^* |
| --- | --- | --- | --- | --- | --- |
|  | Low SES | High SES |  |  |  |
| Acceptability | 4.54 (1.41) | 5.41 (1.30) | 58.26 | <.001 | .34 |
| Practicability | 4.04 (1.47) | 5.1 (1.38) | 86.33 | <.001 | .43 |
| Effectiveness | 3.74 (1.19) | 4.70 (1.31) | 87.56 | <.001 | .43 |
| Affordability | 3.95 (1.51) | 5.85 (1.30) | 140.53 | <.001 | .55 |
| Side effects | 2.71 (1.40) | 2.70 (1.55) | .00 | .957 | .00 |
| Equity | 3.52 (1.33) | 3.98 (1.42) | 17.93 | <.001 | .14 |

Interaction effects between **Type of App** and **SES for Willingness (Table3)** and the **Type of App** and **SES for APEASE criteria of Acceptability, Practicability, Effectiveness, and Affordability (Tables 4-7)**

**Table 3.** Simple main effects analyses results for **Willingness** following an interaction between the Type of App & SES

| Patients’  SES | | App type | *M(SD)* | *p* | | | |
| --- | --- | --- | --- | --- | --- | --- | --- |
|  |  | |  | Prevention | Self- management | | Healthcare |
|  |  | |  |  |  |  | |
| Low | Prevention | | 4.66 (1.77) | - |  |  | |
|  | Self-management | | 5.23 (1.59) | .003 | - |  | |
|  | Healthcare | | 4.76 (1.81) | .01 | 1 | - | |
|  |  | |  |  |  |  | |
| High | Prevention | | 5.42 (1.47) | - |  |  | |
|  | Self-management | | 5.61 (1.44) | .204 | - |  | |
|  | Healthcare | | 5.12 (1.66) | .140 | < .001 | - | |

**Table 4.** Simple main effects analyses results for criteria of **Acceptability** following an interaction between the Type of App & SES

| Patients’  SES | App type | *M(SD)* | *p* | | |
| --- | --- | --- | --- | --- | --- |
|  |  |  | Prevention | Self-management | Healthcare |
|  |  |  |  |  |  |
| Low | Prevention | 4.41 (1.77) | - |  |  |
|  | Self-management | 5.01 (1.73) | < .001 | - |  |
|  | Healthcare | 4.19 (1.63) | .627 | < .001 | - |
|  |  |  |  |  |  |
| High | Prevention | 5.43 (1.46) | - |  |  |
|  | Self-management | 5.66 (1.47) | .071 | - |  |
|  | Healthcare | 5.13 (1.59) | .079 | < .001 | - |

**Table 5.** Simple main effects analyses results for criteria of **Practicability** following an interaction between the Type of App & SES

| Patients’  SES | | App type | *M(SD)* | | *p* | | | |
| --- | --- | --- | --- | --- | --- | --- | --- | --- |
|  |  | | |  | | Prevention | Self-management | Healthcare |
|  |  | | |  | |  |  |  |
| Low | Prevention | | | 3.78 (1.84) | | - |  |  |
|  | Self-management | | | 4.39 (1.66) | | < .001 | - |  |
|  | Healthcare | | | 3.94 (1.65) | | .896 | . 002 | - |
|  |  | | |  | |  |  |  |
| High | Prevention | | | 5.14 (1.59) | | - |  |  |
|  | Self-management | | | 5.27 (1.53) | | .729 | - |  |
|  | Healthcare | | | 4.88 (1.59) | | .173 | .005 | - |

**Table 6.** Simple main effects analyses results for criteria of **Effectiveness** following an interaction between the Type of App & SES

| Patients’  SES | App type | *M(SD)* | *p* | | |
| --- | --- | --- | --- | --- | --- |
|  |  |  | Prevention | Self-management | Healthcare |
|  |  |  |  |  |  |
| Low | Prevention | 3.33 (1.54) | - |  |  |
|  | Self-management | 4.21 (1.43) | < .001 | - |  |
|  | Healthcare | 3.68 (1.54) | .091 | < .001 | - |
|  |  |  |  |  |  |
| High | Prevention | 4.68 (1.49) | - |  |  |
|  | Self-management | 4.96 (1.53) | .029 | - |  |
|  | Healthcare | 4.46 (1.57) | .366 | < .001 | - |

**Table 7.** Simple main effects analyses results for criteria of **Affordability** following an interaction between the Type of App & SES

| Patients’  SES | App type | *M(SD)* | *p* | | |
| --- | --- | --- | --- | --- | --- |
|  |  |  | Prevention | Self-management | Healthcare |
|  |  |  |  |  |  |
| Low | Prevention | 3.77 (1.85) | - |  |  |
|  | Self-management | 4.11 (1.70) | .120 | - |  |
|  | Healthcare | 3.96 (1.67) | .575 | .634 | - |
|  |  |  |  |  |  |
| High | Prevention | 6.02 (1.35) | - |  |  |
|  | Self-management | 5.84 (1.54) | .224 | - |  |
|  | Healthcare | 5.69 (1.43) | .004 | .542 | - |
